# Bio-Inspired Active Skins for Surface Morphing

**DOI:** 10.1038/s41598-019-55163-1

**Published:** 2019-12-09

**Authors:** Yujin Park, Gianmarco Vella, Kenneth J. Loh

**Affiliations:** 1 University of California-San Diego, Materials Science and Engineering Program, La Jolla, CA USA; 2 University of California-San Diego, Department of Structural Engineering, La Jolla, CA USA; 3 Active, Responsive, Multifunctional, and Ordered-materials Research (ARMOR) Laboratory, La Jolla, USA

**Keywords:** Materials for devices, Electrical and electronic engineering

## Abstract

Mechanical metamaterials that leverage precise geometrical designs and imperfections to induce unique material behavior have garnered significant attention. This study proposes a Bio-Inspired Active Skin (BIAS) as a new class of instability-induced morphable structures, where selective out-of-plane material deformations can be pre-programmed during design and activated by in-plane strains. The deformation mechanism of a unit cell geometrical design is analyzed to identify how the introduction of hinge-like notches or instabilities, versus their pristine counterparts, can pave way for controlling bulk BIAS behavior. Two-dimensional arrays of repeating unit cells were fabricated, with notches implemented at key locations throughout the structure, to harvest the instability-induced surface features for applications such as camouflage, surface morphing, and soft robotic grippers.

## Introduction

**Figure 1:**
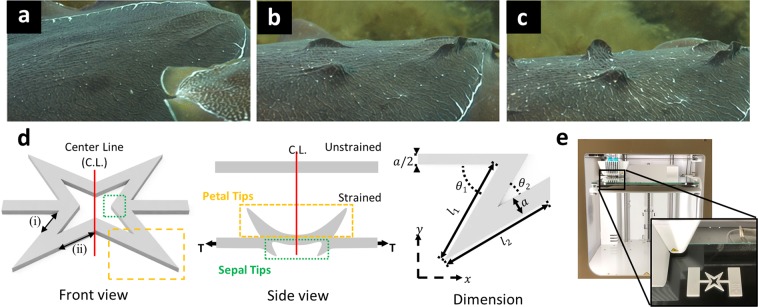
Bio-Inspired Active Skin (BIAS). (**a**–**c**) The Sepia Officinalis displays dynamic skin texture morphing (2D to 3D) from smooth to partially expressed to fully expressed papillae. Skin texture transformation through papillae expression takes approximately 2 s. Figures are reprinted with permission from Springer Customer Service Centre GmbH: Springer, Journal of Comparative Physiology A: Cuttlefish use visual cues to control three-dimensional skin papillae for camouflage, Justine Allen, Lydia M. Ma¨thger, Alexandra Barbosa, Roget T. Hanlon, COPY- RIGHT (2009) (**d**) A rendering (slanted view) and side view of the BIAS star geometry configurations are illustrated with the box showing one of the “arms” of the star geometry and labelled with dimensions: *l*_1_ = 8.486 mm, *l*_2_ = 9.353 mm, *a* = 1.5 mm, *θ*_1_ = 60°, and *θ*_2_ = 30°. (**e**) An Ultimaker 3 + 3D printer was utilized for fabricating all the BIAS star geometries. The zoomed-in picture shows a printed BIAS star unit cell with gripping tabs at both ends for tensile testing.

Nature has shown that some of its creatures can dynamically morph their skin texture to adapt to their ever-changing surroundings. Coleoid cephalopods, namely octopus and cuttlefish, are widely studied examples. They are capable of changing their skin from a smooth to a jagged texture, on-demand and reversibly, for signaling, hunting, and camouflage^1^. In cuttlefish, both the concentric and horizontal erector muscles, which exist in the initially smooth two-dimensional (2D) soft-tissue, contract at discrete locations throughout the cephalopod’s skin to exhibit protruding three-dimensional (3D) surface “bumps” or papillae (Fig. 1a–c). This mechanism is known as muscular hydrostat^1–4^. This ingenious ability of cephalopods to continuously morph their surface texture is direct evidence that their skin is active and multifunctional.

These unique morphing skins found in nature have motivated studies focused on designing and architecting bio-inspired materials that exhibit functionalities such as auxetic behavior^5–7^, enhanced stretch-ability^8^, negative thermal expansion^9,10^, and swelling properties^11,12^. A common mechanism that these carefully structured materials leverage is to excite instabilities within the structure to generate the desired functionalities. More recently, simple harnessing of instabilities through carefully perforated elastic sheets demonstrated its effectiveness as a mechanism for controllable friction properties of a surface^13^. Such architected materials, for example, linear-elastic materials that display non-linear response, can be employed for designing robots^13,14^, actuators^15^, stretchable electronic devices^16^, and shape morphing sheets^17,18^. However, many of the unit cell designs of architected materials are defined at the onset to satisfy just a single property, effectively excluding any possibilities of multifunctionality.

This study presents a Bio-Inspired Active Skin (BIAS), which is a 2D architected material that exhibits local and/or global programmable, rapid, and reversible, out-of-plane surface texture morphing when actuated by in-plane tension. In particular, BIAS exploits the tensile response of a preconceived auxetic unit cell geometrical design that exhibits 2D to 3D deformations when uniaxial tension is applied. By introducing instabilities or notches at judiciously chosen locations within the unit cell, we can effectively control the directionality of out-of-plane deformations, which has broad application possibilities (e.g., camouflage and small- to large-area gripping). Although the BIAS design presented in this work focuses on a specific unit cell pattern, the design principles and material mechanisms are applicable to a multitude of unit cell geometries, which can be designed and pre-programmed to achieve desirable functionalities.

## Results

### BIAS unit cell geometry

The star-shape geometry of our BIAS (Fig. 1d) is a modified version of a preconceived pattern, which was first reported as part of re-entrant cell geometries displaying a negative Poisson’s ratio^19^. The star-shape geometry has a square-symmetrical configuration and has attractive mechanical and physical properties, such as low weight, high strength, and high energy absorption^20^. Due to its simple geometrical configuration and exceptional abilities, it was adopted as static inclusions for modeling fiber-reinforced honeycomb composites^21^, as well as in dynamic studies for wave propagation^22^, band gap^23^, and acoustic super-lens design^20^. Yet, to the best of our knowledge, no investigations on harnessing the load-induced mechanical instabilities produced by the star-shaped pattern have been reported.

Interestingly, the star geometry exhibits mechanical instabilities triggered by uniaxial tension when replicated as an originally flat, elastic substrate below a certain threshold thickness. In the unstrained condition, the star geometry is flat. The geometry undergoes reversible shape morphing with each of the four “arms” of the star protruding out-of-plane when acted upon by uniaxial tension (Fig. 1d). Under uniaxial strain, buckling-induced instabilities of the star geometry trigger out-of-plane shape morphing of jagged features. For simplification, in the deformed state, we differentiate between star geometry arms (calling these petal tips, which are boxed in yellow in Fig. 1d) versus the arrowhead-like tips at the inner junction of two adjacent arms (calling these sepal tips, which are boxed in green). Furthermore, when the star unit cells are interconnected to form arrays, discrete out-of-plane surface morphing of each unit cell results in global texture morphing of the surface with resemblance to that of cephalopods. Figure 1e presents the 3D-printed unit cell star geometry fabricated with tabs for mounting in a load frame.

### Deformation mechanism of BIAS

**Figure 2:**
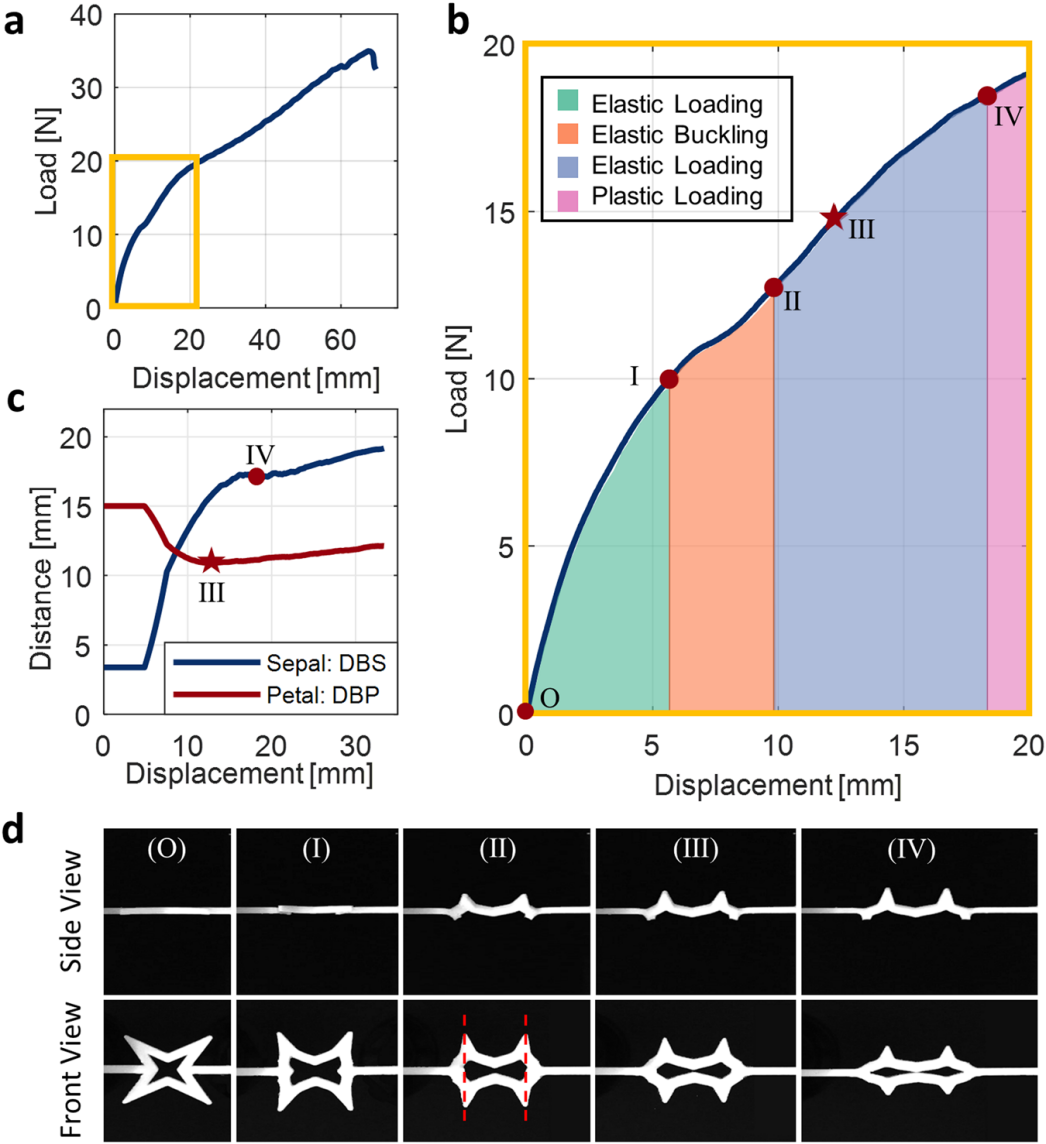
Deformation mechanism of BIAS. (**a**) The load-displacement curve of a 1.5-mm-thick unit BIAS star geometry under uniaxial tension is plotted. (**b**) The elastic deformation zone is highlighted. Each deformation region is segmented through regression analysis, and points corresponding to changes in BIAS behavior are marked. (**c**) The distances between petal tips and sepal tips throughout the deformation of a unit cell star geometry are plotted. Petal tips keep moving towards each other until point III, which is the minimum distance between petal tips. Point III is also marked in (**b**). (**d**) Visual insight of the deformation behavior of the unit cell star geometry at each of the points of interest (from O to IV) is presented using video collected during experimental testing.

The stress-strain deformation mechanism of a single BIAS star geometry that is 1.5-mm-thick subjected to uniaxial tensile loading is depicted in Fig. 2. Unlike a typical stress-strain curve, where elastic deformation (linear) is uniform and is rapidly followed by plastic deformation (nonlinear), the elastic deformation region exhibited by the star geometry appears to be more intricate and segmented (Fig. 2b). The distances between sepal (DBS) and petal tips (DBP) post-buckling are measured for characterizing BIAS behavior and are presented as a performance indicator in Fig. 2c. To elucidate the mechanisms responsible for deformation, we also present in Fig. 2d pictures of the mechanical response of a unit cell under different magnitudes of applied tensile strains.

In the initial stage of elastic loading (i.e., green-shaded region delimited by points O-I in Fig. 2b,d), the deformation of the BIAS star pattern remains in-plane. During elastic loading, the ends of the sepal tips move in parallel with the direction of applied tensile strains until θ_1_ reaches 90° (Fig. 1d).

Following elastic loading, the stress-strain curve transitions to become highly nonlinear (orange-shaded region in Fig. 2b). Effectively, point I corresponds to the critical buckling point of the star geometry as it delineates the start of out-of-plane buckling of the sepal and petal tips. Continued applied uniaxial tension induces outward movements of the sepal tips and results in localized compressive stresses in the angled lateral elements meeting at the sepal tip (Fig. 1d(i)). These compressive stresses induce out-of-plane buckling and emulate a seesaw-like behavior of the petal and sepal tips. Effectively, sepal tips are always pushed in the direction opposite to that of the out-of-plane deformation of the petal tips to accommodate the new equilibrium configuration of the BIAS star geometry. Nonlinear behavior ends when the ends of the sepal tips and petal tips are aligned in Fig. 2d(II).

Thereafter, the stress-strain curve regains linearity (blue-shaded region in Fig. 2b), since buckling has occurred and that there are no additional out-of-plane movements. From points II to III in Fig. 2d, the seesaw-like behavior enlarged the gathering of the petal tips, where this response is only governed by the elongation of the BIAS’ side supporting beams (Fig. 1d(ii)). Thus, DBP reaches the minimum at point III, indicating full out-of-plane deformation or deployment of the geometry as shown in Fig. 2c. After point III, the seesaw-like behavior is attenuated, and the petal tips move away from one another like the sepal tips. Finally, when the curve reaches point IV, plastic deformation occurs (i.e., magenta-shaded region in Fig. 2b).

### Mechanical performance of BIAS

**Figure 3:**
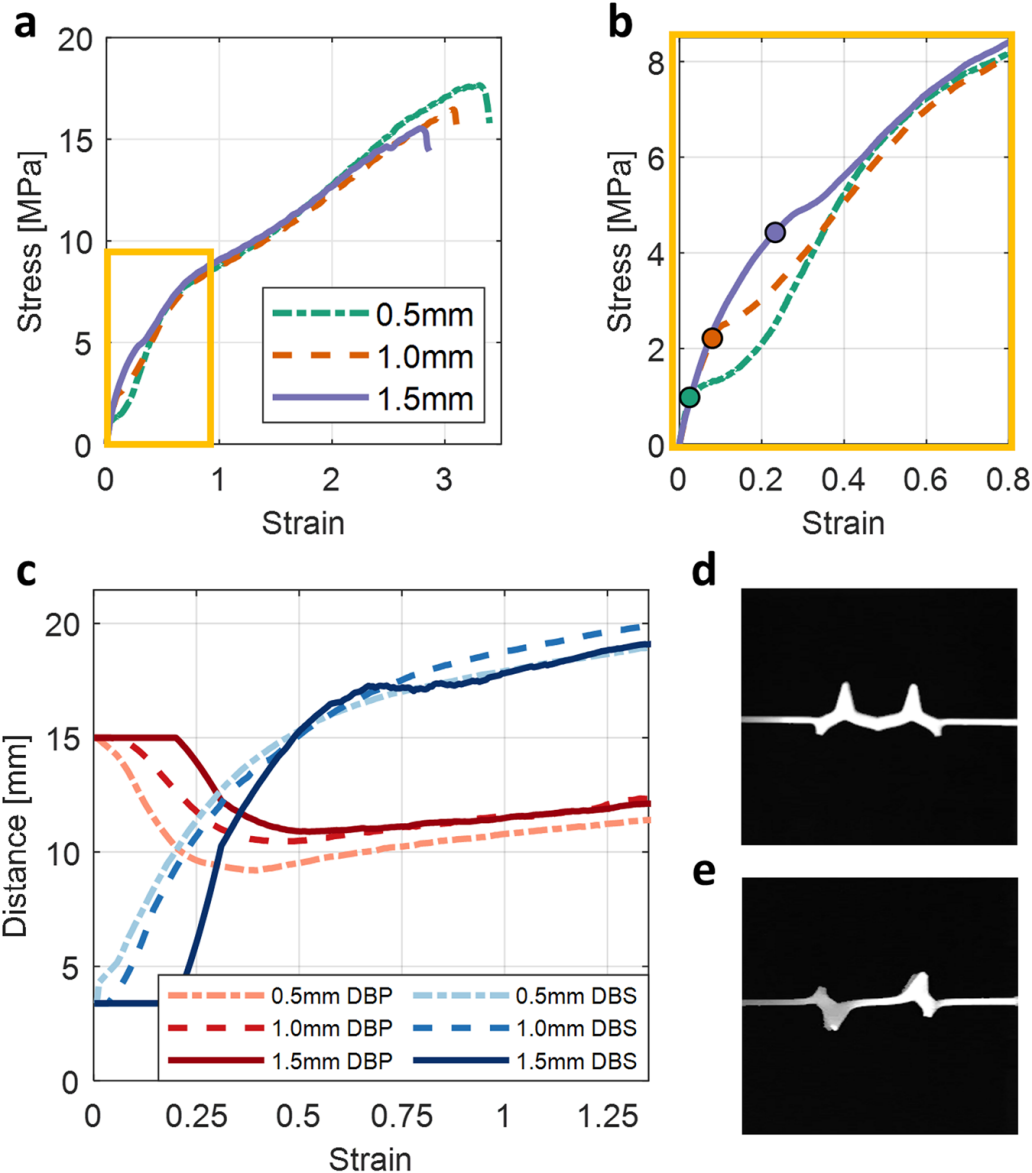
Effect of substrate thickness on the mechanical behavior of a unit cell star geometry. (**a**–**c**) The mechanical response of BIAS with 0.5, 1.0, and 1.5 mm substrate thicknesses is compared. (**a**) Uni-axial stress-strain responses of the star geometry with 0.5, 1.0, and 1.5 mm substrate thicknesses are overlaid. (**b**) The elastic deformation zone of the stress-strain curve (yellow box in A) is highlighted. The critical buckling point for each star geometry is also marked. The curves diverge after the critical buckling point and then overlay each other once elastic buckling occurs. (**c**) The measured distances between petal (DBP) and sepal (DBS) tips throughout the entire deformation process are overlaid. DBP and DBS in the initial or pristine state is 15 mm and 3.38 mm, respectively. (**d**,**e**) Unidirectional and random out-of-plane buckled configurations of the star geometry under applied uniaxial strains were observed during testing.

Figure 3a captures the mechanical behavior of the BIAS star geometry and the effects of different thicknesses under applied uniaxial tension. As substrate thickness decreases, Fig. 3b shows that the critical buckling point (or the initiation of out-of-plane shape morphing) shifts leftwards and downwards along each line. Otherwise, the initial stress-strain responses of different thickness BIAS stars superimpose and are governed by the linear-elastic response of the material used. Since critical buckling load is linearly proportional to bending stiffness and decreases as the thickness of the substrate is reduced, there is an energetical benefit in using lower substrate thicknesses as this results in lower applied uniaxial tension to start and attain full out-of-plane shape morphing. Figure 3c also confirms that DBP changes more dramatically when star thickness and bending stiffness are reduced.

In theory, the buckling-induced, out-of-plane, shape morphing orientations of the star geometries are highly dependent on load perturbations and material inhomogeneities regardless of their thickness. Despite the advantages of reducing bending stiffness, 3D-printing of star geometries on thinner substrates make them even more susceptible to process-related phenomena and material inhomogeneities. This effect was witnessed during our experimental study, where 0.5- and 1.0-mm-thick star geometries produce random, out-of-plane, petal tip deformation directions (Fig. 3d,e).

### Programmable BIAS through imperfections

**Figure 4:**
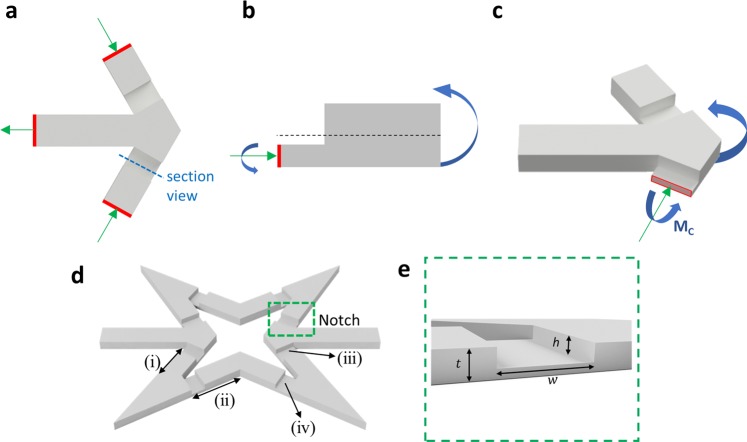
Deterministic and programmable deformation of the BIAS star geometry. (**a**) The top view of a rendered sepal tip is shown, where the horizontal supporting beam connects the two angled lateral elements to form the sepal tip. (**b**) The side view of a portion of the sepal tip is shown. Imperfections, or notches, shift the neutral axis downwards and forces out-of-plane deformations to occur in a deterministic and programmable direction. (**c**) A rendering of a sepal tip’s out-of-plane deformation under uniaxial tension is illustrated. (**d**) A rendering of the notched unit cell star geometry is shown, where (i) and (ii) represent the beams of the BIAS that meet at the sepal tips and supporting beam, respectively. (iii) and (iv) are the notches induced to control buckling direction and to maintain static equilibrium. (**e**) The thickness, *t*, depth, *h*, and width, *w* of notches in the star geometry are illustrated.

As a means to control and harness the buckling-induced instabilities of the BIAS star geometry, we introduced geometrical imperfections or notches at key locations throughout the deformation mechanism of the structure. When the BIAS is strained in tension, sepal tips are under localized compressive stresses to maintain equilibrium, which are shown by the green arrows in Fig. 4a. These notches shift the neutral axis locally and asymmetrically (Fig. 4b,c), thereby generating a bending moment (blue arrows) that drives the out-of-plane motion of the sepal tips. Such purposefully designed geometrical imperfections overpower the undesirable effects of material inhomogeneities to enable directional control of out-of-plane buckling-induced deformations.

Therefore, geometrical imperfections were judiciously placed at the start/end of each arm of the star geometry as shown in Fig. 4d,e. Four notches, as shown in Fig. 4d(iii), were introduced at the base of the sepal tips, where compressive stresses are localized. Applied in-plane tension to the supporting beams (Fig. 4d(ii)) results in compressive stresses in the arms with notches and ultimately results in localized buckling around Fig. 4d(iii). In addition, four additional notches (Fig. 4d(iv)) were placed next to the petal tips. As applied tension induces separation of the sepal tips and the buildup of internal bending moments near notches shown in Fig. 4d(iii), the notches in Fig. 4d(iv) drive out-of-plane deformations of the petal tips to maintain static equilibrium.

**Figure 5:**
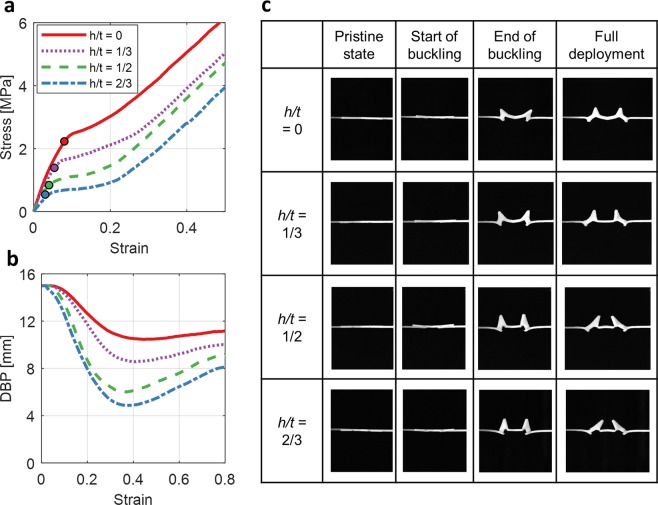
Effect of notches on the mechanical behavior of a 1.0-mm-thick unit cell. (**a**) The mechanical response of notched BIAS for *h/t* = 1/3, 1/2, and 2/3 are overlaid. (**b**) The measured distance between petal tips throughout the deformation process is plotted. (**c**) Image frames extracted from the video taken during tensile testing of the notched BIAS star geometry shows its deformation behavior at each of the points of interest (from points O to III in the deformation mechanism).

The effect of notch height (*h*) to thickness (*t*) ratio (i.e., *h/t* = 1/3, 1/2, and 2/3 while fixing width, *w* = 1.0 mm) on the mechanical behavior of the BIAS star geometry was also investigated. The stress-strain response of a 1.0-mm-thick star geometry was directly compared to its respective unnotched (*h/t* = 0) configuration (Fig. 5a–c). Even the introduction of the shallowest notch (*h/t* = 1/3) resulted in reduced critical buckling stress (or load) by ~ 40% (Fig. 5a). Furthermore, increasing normalized depths of the notches further decreased critical buckling load (Fig. 5a) while improving out-of-plane deformations of the star geometry, which is quantified by DBP (Fig. 5b). As expected, the notched star geometry with *h/t* = 2/3 resulted in the greatest petal tip deformation at the least amount of tensile stresses applied.

**Figure 6:**
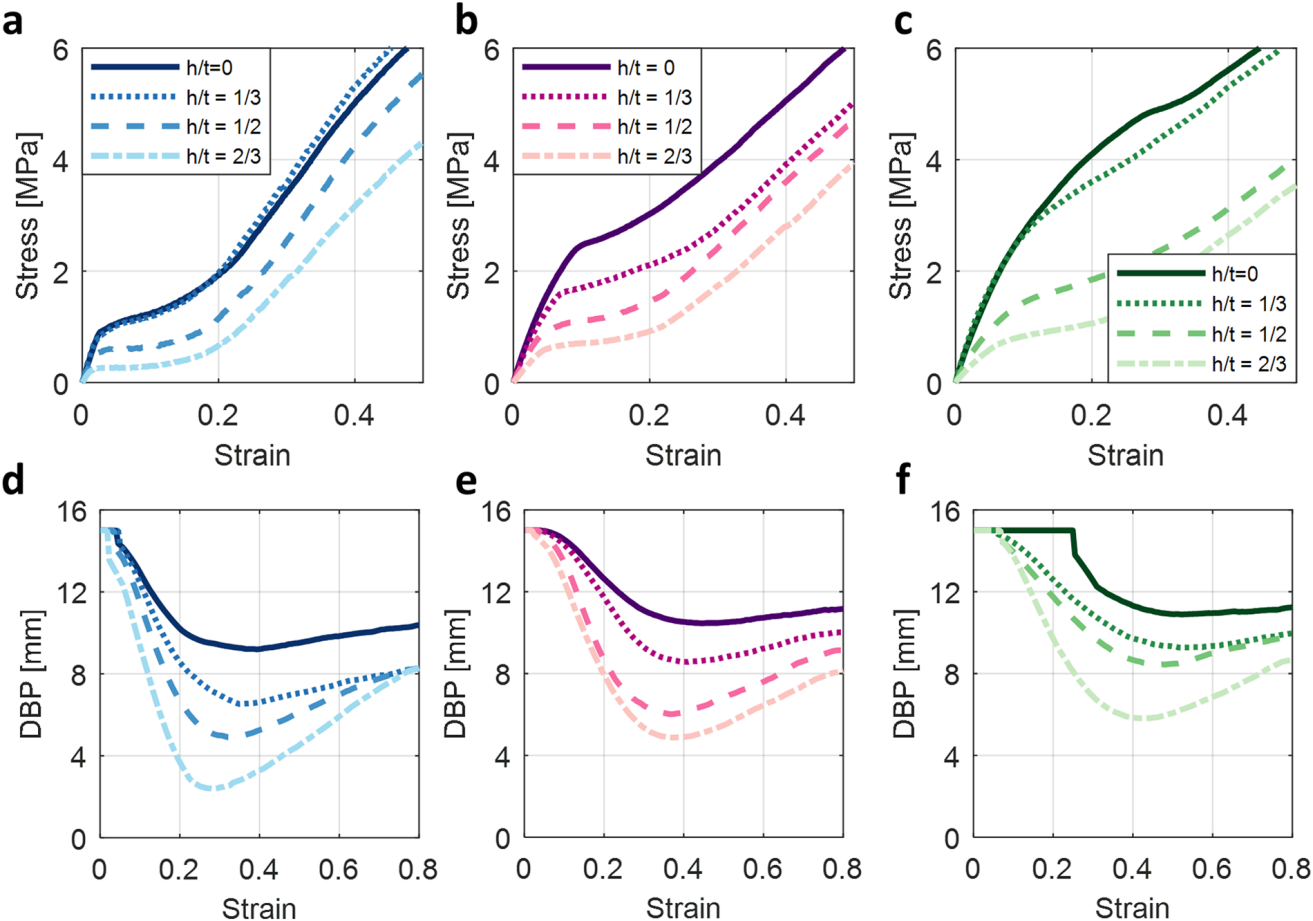
Mechanical behavior of a notched star geometry unit cell with *t* = 0.5, 1.0, and 1.5 mm and *h/t* = 1/3, 1/2, and 2/3. (**a**–**c**) The mechanical response of notched star patterns in ascending order of *t* (left to right) are presented. (**d**–**f**) The corresponding measured distance between petal tips throughout the deformation process is also overlaid to compare BIAS behaviors.

Figure 6 exhibits the stress-strain response of notched BIAS unit cells in ascending order of *t* (*i.e., t* = 0.5, 1.0, and 1.5 from left to right). Overall, the judicious placement of notches throughout the star geometry shifts the critical buckling point downwards and leftwards, which reduces activation stress and strain needed to trigger buckling-induced out-of-plane deformations. All the conditions appeared to follow this trend, except for specimens with *t* = 0.5 mm and *h/t* = 2/3. These specimens (being the thinnest and with the deepest notch depths) were most susceptible to fabrication-related imperfections and inhomogeneities. This observation was leveraged for the selective actuation of BIAS. Furthermore, the DBP results clearly showed that greater notch depths led to larger changes in DBP, with the unnotched (*h/t* = 0) resulting in the least amount of change in DBP.

### Reversibility of BIAS

**Figure 7:**
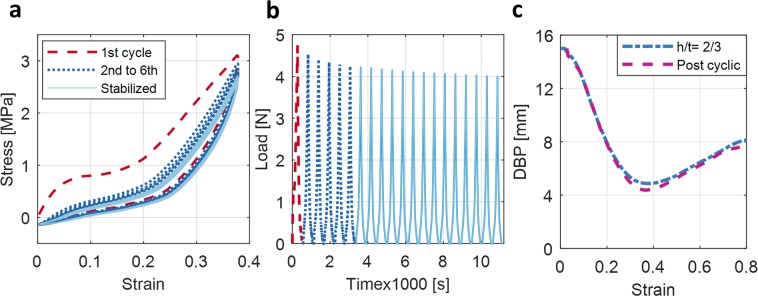
Mechanical response of a BIAS unit cell subjected to uniaxial tensile cyclic strains. (**a**) Stress-strain hysteresis curves are obtained during tensile cyclic tests. (**b**) The plot shows the applied cyclic load time history necessary to attain full BIAS deployment. (**c**) DBP plots corresponding to the first cycle of tensile loading and post-cyclic testing show comparable behavior.

Buckling of materials in the elastic domain is reversible and repeatable. To study the reversibility of BIAS, the star geometry was tested under displacement-controlled tensile cyclic loading. The unit cell star geometry was cyclically strained from 0 to approximately minimum DBP (i.e., equivalent to point III in Fig. 2b) to attain full BIAS deployment. All tensile cyclic tests were conducted using specimens that were 1.0-mm-thick and *h/t* = 2/3. Figure 7a overlays the stress-strain hysteresis response of a representative specimen subjected to tensile cyclic loading. First, the stress-strain response of the 1^st^ cycle is consistent with the results presented earlier in Fig. 5a. Evidence of slight plastic deformation and/or creep is seen in this case, where compressive stresses are induced to return the specimen back to its pristine state.

Second, the next five cycles of loading (2^nd^ to 6^th^) suggest stress-softening of the star geometry, where maximum stress at peak tensile strain decreases progressively with each additional cycle of loading. Finally, a stabilized hysteresis loop is obtained after six loading cycles. Figure 7b illustrates the decrease in applied load to attain full deployment of the notched 1.0-mm-thick BIAS star pattern following cyclic loading. Despite the change in its stress-strain response, out-of-plane deformation behavior post-cyclic loading is comparable to its pristine (non-cycled) specimen as shown in Fig. 7c, thus clearly highlighting the reversibility and repeatability of the shape morphing properties of the BIAS star geometry. Overall, out-of-plane deformation is triggered by applying low magnitudes of strain, and BIAS can respond to suddenly applied strains and in a controlled manner^24^.

### Versatility of BIAS

**Figure 8:**
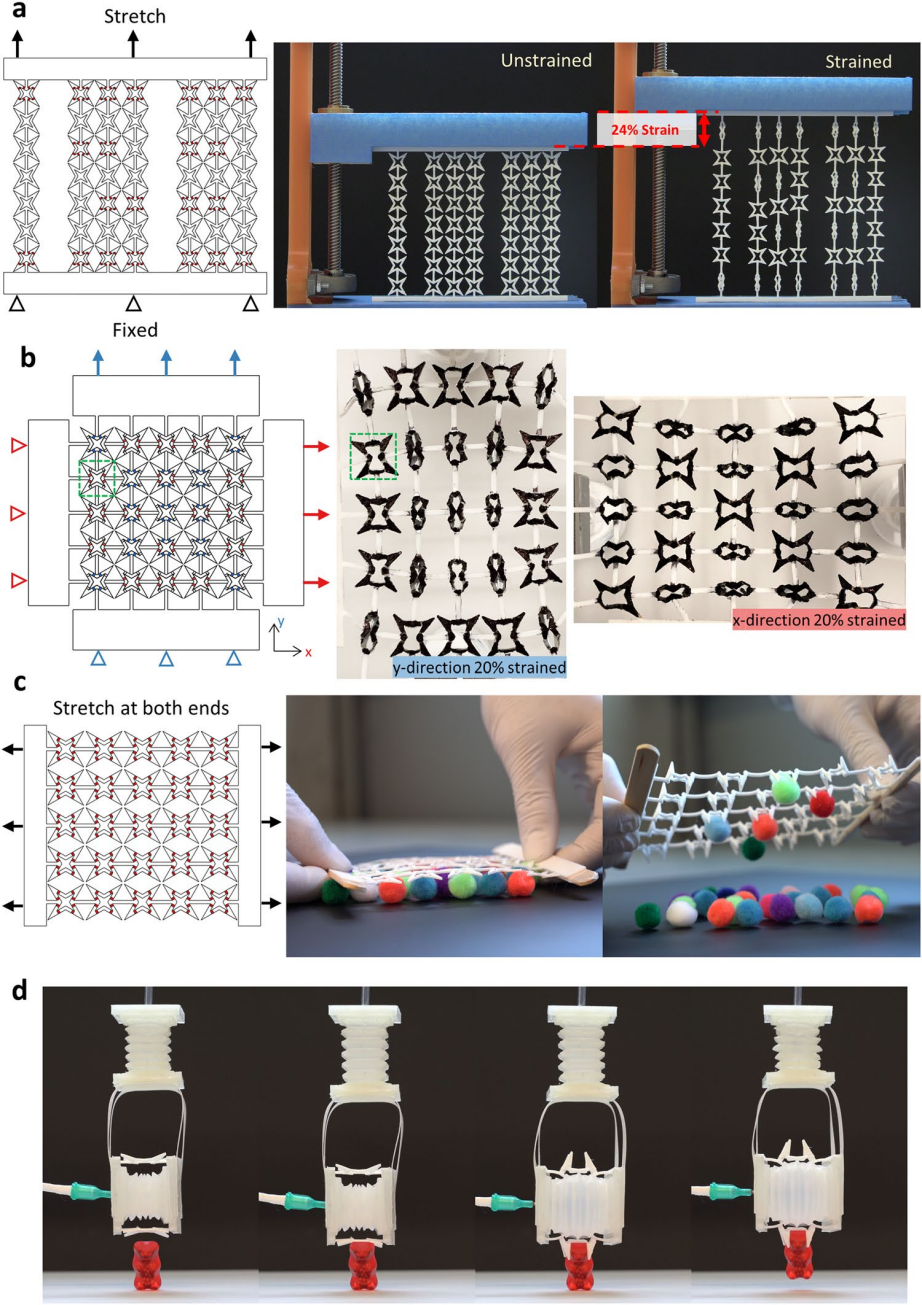
Designs of BIAS arrays for camouflage and large-area and small-area gripping. (**a**) A BIAS array was pre-programmed to reveal the numbers “1 2 3” by selectively actuating the interconnected star array at discrete locations once uniaxial tensile strain was applied. (**b**) The BIAS array was interconnected in the vertical and horizontal directions. The pattern was designed to show an “O” when strained vertically in tension and an “X” when strained horizontally in tension. (**c**) An array was designed for large-area gripping of multiple soft fur balls (~15 mm diameter) by applying uniaxial strains to its vertical edges. (**d**) A single BIAS unit cell star was used for gripping a gummy bear (~4 g). The soft crane includes two soft, linear, accordion pneumatic actuators. Uniaxial tensile strain was applied to the single gripper by inflating and expanding the soft accordion actuator.

The versatility of the methodology for harnessing mechanical instabilities through geometrical imperfections presented thus far is shown in Fig. 8. To pre-program BIAS for different functionalities, the design approach is to include, omit, or alternate the placement of notch designs at judiciously chosen locations in arrays of 3D-printed unit cell star geometries. First, a 7 × 7 array of interconnected star geometries was designed and printed to display programmable and localized actuation (Fig. 8a). During array design, stars highlighted in red were notched, while all others were unnotched. Uniform, uniaxial tension was applied to stretch the array in the vertical direction while keeping the bottom fixed. Because unnotched stars require larger strains to induce out-of-plane deformations, the induced strains acting on the pre-programmed BIAS resulted in controlled shape morphing to display an embedded “1 2 3” pattern (Fig. 8a and see Supplementary Movie S1). It should be mentioned that the alternation of notched to unnotched star geometries for successfully displaying an embedded pattern is only valid up until the critical buckling load of the unnotched star geometries. Further straining the array would deploy the entire BIAS pattern.

Second, a 5 × 5 BIAS array of black-colored star geometries was pre-programmed, by interconnecting unit cells in both the vertical and horizontal directions, so that two different embedded patterns (i.e., “O” and “X”) can be revealed depending on the direction of applied loading (Fig. 8b). Unlike the “1 2 3” pattern, all the star unit cells notched in this BIAS pattern and the notch locations in each unit cell are different. Notches placed near the sepal tips on vertically interconnected lines of stars highlighted in blue were designed to display an “O” pattern, whereas notches in stars highlighted in red were placed near the sepal tips on horizontally interconnected lines for revealing an “X” pattern. Moreover, notches near the petal tips hindered out-of-plane deformation of the petal tips more so than notches near the petal tips located on the beams perpendicular to applied tension (i.e., green-dashed box). The pre-programmed BIAS array in Fig. 8b strained to 20% shows controlled morphing to display an embedded “O” when subjected to uniaxial tension in the vertical direction and displayed an embedded “X” when strained in tension in the horizontal direction.

Third, the out-of-plane shape morphing behavior of star geometries was harnessed for gripping. A 5 × 5 BIAS array of interconnected notched stars throughout the entire array was 3D-printed and demonstrated for large-area gripping (Fig. 8c and see Supplementary Movie S2). The BIAS array was placed over randomly distributed fur balls (~1 g each) and then stretched manually to engage the star grippers for grabbing multiple objects at once. Unique to using BIAS for gripping is the use of in-plane tension as the actuation mechanism. This is in contrast to most grippers that require the direct application of compressive forces on two opposing surfaces (or fingers) to restrict the motion of an object trapped in between. One can observe in Fig. 8c that some stars folded in a random (or incorrect) direction despite the use of notched stars. These random pattern deployments in different directions (despite the presence of notches) were a result of manually stretching the star array, where rotations and nonuniform tensile strains were inadvertently applied.

Last, the coupling of an accordion-like, soft, linear actuator with BIAS stars mounted on its opposite faces enabled the creation of a soft robotic claw crane (Fig. 8d and see Supplementary movie S3)^25^. Pneumatically actuating the soft accordion caused it to extend horizontally, thereby applying uniform tensile strains to the BIAS and triggering out-of-plane folding of the star arms. An additional linear accordion actuator was connected perpendicular to the “soft claw” for raising and lowering the BIAS soft robotic claw (see Supplementary Fig. S1). The demonstration successfully validated that the BIAS claw could lift a gummy bear that weighed ~4 g. Overall, the demonstration examples shown in Fig. 8 confirm that BIAS arrays can be pre-programmed and actuated for multiple engineering functionalities.

## Discussion

In conclusion, we present a Bio-Inspired Active Skin, which exhibits programmable, reversible, rapid, and on-demand texture morphing, that is actuated by applied in-plane tensile strains. BIAS leverages buckling-induced instabilities in the form of notches introduced at judiciously chosen locations in a star geometry. The optimal notch depth and substrate thickness combination of BIAS were found by characterizing their deformation mechanism and the mechanical response of unit cell star geometries subjected to uniaxial tensile loading. In addition, BIAS unit cells and arrays were fabricated using a commercial-off-the-shelf benchtop 3D printer, which further demonstrates their scalability and the effectiveness of a simplified design methodology. The design approach followed in the making of the BIAS presented herein can be extended to a multitude of existing architected geometries, enlarging their field of applications and hence their multifunctionality. Further developments of the BIAS array aim at deriving an inverse design methodology to inform optimal placement of geometrical instabilities that can reliably produce desirable system shape morphing.

## Methods

### 3D Printing of BIAS

Each unit cell star geometries and interconnected arrays were fabricated using an Ultimaker 3, which is a commercial fused deposition modeling (FDM) 3D printer. Thermoplastic polyurethane (TPU 95 A) filament (Dynamism Inc.) was used to 3D-print all of the star geometries and arrays. The geometries were designed in Autodesk Fusion 360 (2018) with dimensions found in Fig. 1d. The 3D models (.stl) of the star geometries and all interconnected arrays were loaded in Ultimaker Cura 3.3 and fed to the 3D printer for fabrication.

### Tensile and tensile cyclic testing of BIAS

Uniaxial tensile and cyclic tests of the star geometry were performed by mounting each star unit cell in a Test Resource 150 R load frame equipped with a 10 N load cell. The load frame was commanded to stretch the star geometry along the direction of its longitudinal axis from 0 until failure at a constant applied displacement rate of 2 mm/min while simultaneously recording load and displacement using Keysight Benchvue. On the other hand, the reversibility of the BIAS star geometry was characterized by subjecting star geometries (i.e., 1.0-mm-thick and with a normalized notch depth of *h/t* = 2/3) to a 20-cycle tensile load pattern to a maximum strain of 37%. The applied displacement rate was fixed at 2 mm/min, while load, displacement, and time were recorded simultaneously.

## Supplementary information

Supplementary Information

Supplementary Movie S1

Supplementary Movie S2

Supplementary Movie S3
