# Supplementary material for: Bio-Inspired Active Skins for Surface Morphing
Source: Sci Rep. 2019 Dec 9;9:18609. doi: 10.1038/s41598-019-55163-1 (PMC6901544; doi:10.1038/s41598-019-55163-1)
Supplement: Supplementary file 1 — Supplementary Information [file 41598_2019_55163_MOESM1_ESM.pdf]

Bio-Inspired Active Skins for Surface Morphing

Supplementary Information

Yujin Park[1,3], Gianmarco Vella[1,3], and Kenneth J. Loh[1,2,3,*]
[1] University of California-San Diego, Materials Science and Engineering Program, La Jolla, CA, USA
[2] University of California-San Diego, Department of Structural Engineering, La Jolla, CA, USA
[3] Active, Responsive, Multifunctional, and Ordered-materials Research (ARMOR) Laboratory, La Jolla, USA
[*] Corresponding author e-mail: kenloh@ucsd.edu

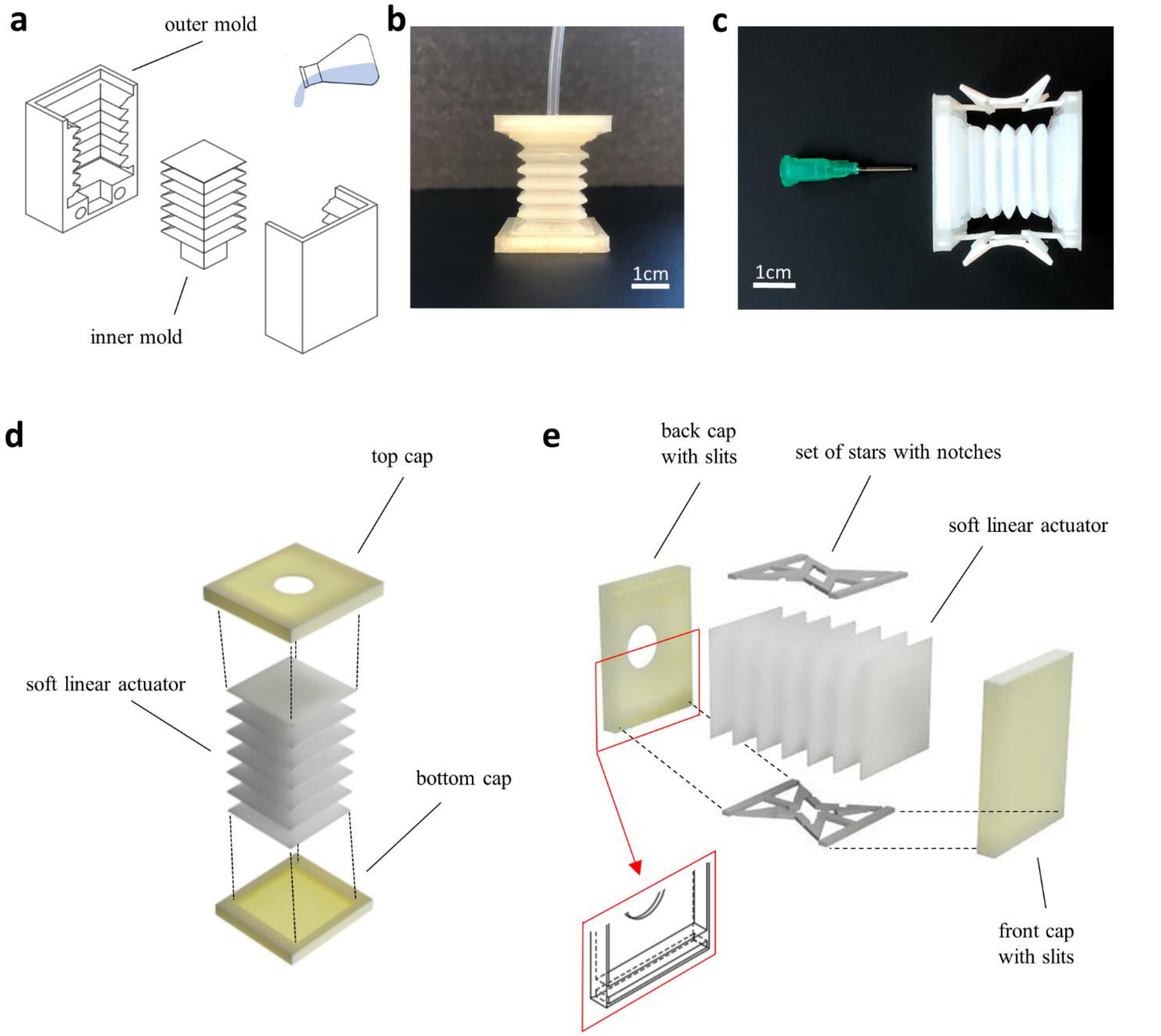

Figure S1: Fabrication of the "accordion" soft linear actuator. a) A schematic of the 3D-printed mold for casting the soft accordion actuator is shown. The accordion-like, soft, linear actuators were made by pouring FX-Pro elastomer (Smooth-On Inc.) into a three-piece polylactic acid mold. The inside of the hollow actuator resembled the outer, accordion-like shape. The open end of the soft actuator was then sealed with additional FX-Pro elastomer, making the actuator airtight. b-c) The soft accordion actuator (for vertical actuation) is connected with a flexible, plastic tube. An 18-gauge syringe needle was inserted in the airtight bottom horizontal actuator and sealed with additional FX-Pro elastomer to avoid any leaks. d-e) Two 3D-printed PLA caps with slits to fit the two BIAS star unit cells were also printed and mounted onto the ends of the soft accordion actuator ("soft crane"). The BIAS star "grippers" were scaled up by 25% versus the ones used for tensile testing and analysis of its deformation mechanism. The stars were inserted in the caps' slits and glued with epoxy (Gorilla Glue). The two vertical and horizontal actuators were connected by means of a 3D-printed TPU 95A strip.
